# A National School of Tropical Medicine and Neglected Infections of Poverty for North America

**DOI:** 10.1371/journal.pntd.0000735

**Published:** 2010-06-29

**Authors:** Peter Hotez

**Affiliations:** Sabin Vaccine Institute and Department of Microbiology, Immunology, and Tropical Medicine, George Washington University Medical Center, Washington, D.C., United States of America

The turn of the 20th century witnessed the creation of the world's first two schools of tropical medicine. The Liverpool School of Tropical Medicine admitted its first student in May of 1899, and the London School of Tropical Medicine, which eventually became the London School of Hygiene & Tropical Medicine, six months later [1]. At the beginning, the two British tropical medicine schools were founded on different principles [1], [2]. The Liverpool School was built to provide medical support for a vigorous shipping trade between Liverpool, one of England's most active ports, and the coasts of Africa, Asia, and the Caribbean [1]. Launched with an initial gift from Alfred Lewis Jones, who made his fortune from shipping interests in West Africa and elsewhere, the majority of the Liverpool School's patients who were admitted to the tropical ward of its affiliated Royal Southern Hospital worked in the shipping trade [1]. From 1899 until the outbreak of World War I a cornerstone of the Liverpool School was expeditions financed by the Suez Canal Company, the Panama Canal Commission, and other overseas shipping concerns [1]. The London School had a somewhat different focus. Established through the force of personality of Sir Patrick Manson (“the father of tropical medicine”), the London School's original mission was to train general colonial medical officers employed in the services of a vast British empire, which included the military and the Indian Medical Service [2]. By the early 1900s, it was estimated that 10% or more of British physicians were employed as overseas practitioners [2].

Many of the other Western European countries with large colonial holdings subsequently created schools or institutes of tropical medicine, including the Bernhard Nocht Institute for Tropical Medicine in Hamburg, Germany (1900), the Institute of Tropical Medicine in Antwerp, Belgium (1905), the Royal Tropical Institute (KIT) in Amsterdam, The Netherlands (1910), and the Swiss Tropical and Public Health Institute in Basel (1943), among others. In addition to training medical practitioners to work overseas, in time both the British schools and European Institutes evolved distinguished and vital programs of biomedical research that led to the discoveries of the life cycles of several important human parasites and many of the drugs and diagnostics for neglected tropical diseases (NTDs) still in use today. Thus, while some of these institutions may have been founded with colonialist intentions, in short order they became international resources for research and development to combat some of the most important NTDs afflicting the world's poor. Today, the same institutions also maintain strong research training programs in the biomedical sciences with excellent track records of trainees who populate institutions in both the North and the South.

Fast forward 100 years, and at the beginning of this new Millennium there is still no fully dedicated school or institute located in North America with the depth and breadth in NTDs research, development, and training, as those maintained by the two British tropical medicine schools or even some of the larger tropical institutes on the European continent. To be sure, there are pockets of great excellence in research and training for selected tropical diseases in the schools of public health at Harvard, Johns Hopkins, Tulane, University of California, Berkeley, University of Washington, and the National Institute of Public Health in Cuernavaca, Mexico. There is also important basic research conducted at the National Institutes of Health and the Institute of Parasitology at McGill; superb public health training at the U.S. Centers for Disease Control and Prevention; and innovation leading to the development of new control tools at product development partnerships in Seattle, Washington; San Francisco, California; and Washington, D.C. However, today in North America there is no comprehensive school or institute for NTDs that hosts a full complement of these activities, especially for multiple tropical infections. Similarly, there is no equivalent entity located in North America that provides extensive training in whole-organism biology for the recognition and manipulation of most of the major NTD parasitic, bacterial, and viral agents or their vectors. Thus it is not clear whether there is a school in North America whose graduates could pass a modern equivalent of the 100-year-old final examination of the London School of Tropical Medicine:

“*Students were asked to describe the methods for demonstrating the Widal Reaction in typhoid and Mediterranean fever and for purifying water on the march; to distinguish between the* Anopheles *and the* Culex *mosquito and the different filarial embryos; to diagnose leprosy, syphilis, lupus and malaria; and to describe the recommended treatments for cholera and Dhobi itch. The laboratory practicum tested for competence and little more. Students were asked to describe the steps to identify an unknown broth in a test tube, to stain blood samples for revealing the malarial parasite, and to identify abnormalities and determine the stage of infection based on microscopic specimens*” ([2] p. 159)

Previously, my colleagues and I recommended that North American schools of public health and medicine pay increased attention to the concept of appropriate technology, referring to innovations “developed, produced, delivered and monitored within a comprehensive framework that takes into account the systems, the individuals, and the community” [3]. Today, we live in the midst of an exciting era in which support for research and development for NTDs is beginning to increase because of the activities of both private foundations, such as the Bill & Melinda Gates Foundation and Wellcome Trust, and the U.S. National Institutes of Health [4]. But in many cases training has not kept pace with the advances in technology, so that in North America there currently does not exist “one stop shopping” where an individual can learn, for example, how to apply the use of Affymetrix chip and deep sequencing technologies toward the development of new NTD diagnostics; how to apply high-throughput drug screening and process development for new NTD drugs and vaccines, respectively; and then how to apply such technologies into global public health practice [4]. Today such training is offered in a fragmented or haphazard manner, or in many cases is totally unavailable across most public health and medical schools in North America. On this basis, I have argued previously that we need a school of appropriate technologies both to train the next generation of global health scientists who can develop these new control tools and to introduce these tools in a manner that is compatible with health systems [4].

In addition to the dearth of training offered in global health–appropriate technologies, I believe that opportunities for learning how to work directly with NTD pathogens are also extremely limited. Life cycles of many parasites and other whole organisms, as well as their vectors, are frequently difficult to maintain in the laboratory. Indeed, the availability of living parasites and organisms in any one particular school or institute in North America for purposes of instruction is often limited to whatever organisms are on hand because of on-site research investigators who study these pathogens through funded programs. The availability of patients with tropical diseases and other clinical material is similarly deficient, and North American schools that provide instruction for health professionals to sit for the certifying examination offered by the American Society of Tropical Medicine and Hygiene often struggle to place students in meaningful clinical experiences.

In response to similar concerns, several North American schools of public health and medicine have launched training partnerships with institutions in low- and middle-income countries. These include important “twinning” opportunities between North American institutions and sister schools in sub-Saharan Africa. Through the Fogarty International Center, the U.S. National Institutes of Health has just announced a Medical Education Partnership Initiative (MEPI) with the President's Emergency Program for AIDS Relief (PEPFAR) to further support such U.S. and African training partnerships [5], which will also offer enormous potential for capacity-building in the region. At the same time, I believe there remains a strong need to have a centralized facility in North America for training in tropical medicine, i.e., one that embraces whole-organism biology of key NTD pathogens, new and appropriate health technologies and their introduction into global public health practice, and clinical tropical medicine. A National School of Tropical Medicine or the equivalent based in North America would address an important gap in training in the region. In addition, through distance learning and clinical activities a National School would in time extend international outreach with other schools and institutes in the South. For instance, a low-cost distance learning program in Spanish and Portuguese would allow for ready partnering with institutions in Latin America, where the burden of NTDs is surprisingly high [6]. A National School in North America would also provide training on parasitic infections and other neglected infections of poverty that were recently revealed to disproportionately affect African American and Hispanic minority populations in the U.S. and Canada [7], [8]. There are multiple solutions for meeting the training needs of the next generation of global public health experts, including the possibility of bringing together outside experts to a neutral site (or rotating university) to offer focused didactic opportunities. However, establishing a centralized and comprehensive National School could also represent one such important piece of the answer.
